# Castration-resistant prostate cancer patient presenting with whole genome doubling with CDK-12 mutation

**DOI:** 10.1186/s12920-022-01178-z

**Published:** 2022-02-19

**Authors:** Yuto Baba, Takeo Kosaka, Hiroaki Kobayashi, Kohei Nakamura, Shuji Mikami, Hiroshi Nishihara, Makoto Nakanishi, Mototsugu Oya

**Affiliations:** 1 Department of Urology, Keio University School of Medicine, 35 Shinanomachi, Shinjuku-ku, Tokyo, 160-8582 Japan; 2 Genomics Unit, Keio Cancer Center, Keio University School of Medicine, Tokyo, Japan; 3 Division of Diagnostic Pathology, Keio University Hospital, Tokyo, Japan; 4 The Division of Cancer Cell Biology, Institute of Medical Science, The University of Tokyo, Tokyo, Japan

**Keywords:** Castration-resistant prostate cancer, Bone metastases, Whole-genome sequencing, Case report

## Abstract

**Background:**

The use of whole-genome sequencing in clinical practice has revealed variable genomic characteristics across cancer types, one of which is whole-genome doubling (WGD), which describes the duplication of a complete set of chromosomes. Yet it is relatively rare in prostate cancer and no such case has ever been reported in Japanese patients.

**Case presentation:**

A 54-year-old patient with prostatic adenocarcinoma with bone and lymph node metastases was started on androgen-deprivation therapy. As the prostate cancer turned castration-resistant, multimodal therapies including taxane- and platinum-based chemotherapy, androgen-receptor antagonist inhibitors, radiotherapy and radium-233 were introduced. Good controls of serum prostate-specific antigen (PSA) level and bone metastases were achieved for more than 13 years since after the initial treatment. During the treatment, a metastatic lymph node biopsy was performed to confirm the tumor histology, and spinal decompression surgery were performed for spinal compression due to lumber vertebral metastases. The immunohistochemical analysis identified PSA and androgen receptor positive tumor cells in both metastatic lesions, while no variable cancer cells were detected in the prostate on second biopsy. Whole-genome sequencing was performed on the biopsied metastatic lymph node in search for another possible treatment and it revealed that the tumor had WGD and CDK12 mutation. The WGD-positive tumor cells contained large and polymorphic nucleus, presumably reflecting on the ploidy abnormality of the chromosomes.

**Conclusions:**

This report is the first case of a Japanese patient presenting with WGD, who survived more than 13 years with multimodal chemotherapies and radiotherapies.

**Supplementary Information:**

The online version contains supplementary material available at 10.1186/s12920-022-01178-z.

## Background

Recently large-scale genomic studies have enabled utilization of whole exome-sequencing (WES), the sequencing of all the protein-coding regions of individual cancer-patient genome, in search for precision therapeutic strategies. Major driver-gene mutations found in this the WES could provide candidates for applicable therapeutic medicine beyond cancer types [1]. Application of genome sequencing in clinical practice has revealed the new genomic characteristics of various cancers. One of them is whole-genome doubling (WGD), the doubling of a complete set of diploid chromosomes, which considered to be a poor prognostic factor or a contributing factor for copy number alterations in certain cancers [2, 3]. On the other hand, the significance of WGD in prostate cancer remains unclear and its presence has not even been reported in the Japanese population. Herein, we present the case of castration-resistant prostate cancer who received multimodal therapies for more than 13 years and eventually developed WGD.

## Case presentation

### Initial diagnosis and treatment

**Fig. 1:**
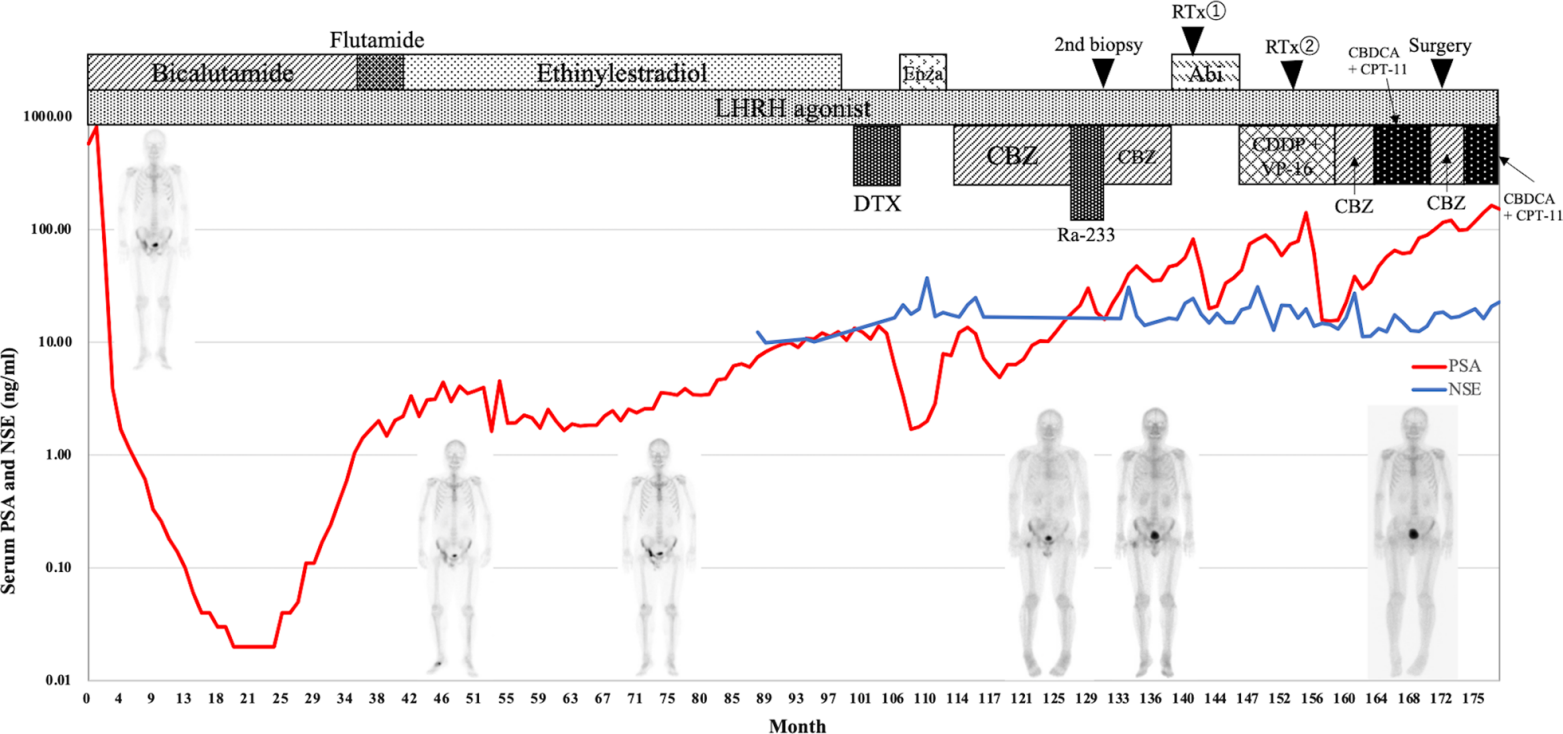
Serum prostate-specific antigen (PSA) and neuron-specific enolase (NSE) levels and chronological treatments. Bone scintigraphy is placed at each time period. Before radium-223 (Ra-223) initiation, a new uptake appeared in the right femoral head. Six courses of Ra-223 did not make a difference in the image, but after platinum-based chemotherapy and twice external beam radiotherapies (EBRTs), a remarkable improvement was observed in the bone scan

A 54-year-old male with an elevated serum prostate-specific antigen (PSA) level (573.9 ng/mL) was referred to our facility. He had no remarkable medical or family histories. Transrectal prostate biopsy revealed prostate cancer in 9 out of 10 cores, with a Gleason score of 4 + 5. Through MRI and computed tomography (CT), a tumor was detected in the right lobe of the prostate, clearly expanding beyond the capsule to the right seminal vesicle; multiple lymphadenopathy and bone metastases were also noted. Thus, he was diagnosed with cT3bN1M1b prostate cancer. At the first diagnosis of prostate cancer, the patient was started on androgen deprivation therapy (ADT) with luteinizing hormone-releasing hormone (LHRH) agonist in combination with antiandrogens such as bicalutamide, flutamide, ethinylestradiol as the first-line therapy, which dramatically decreased his PSA level to a nadir of 0.02 ng/mL after 19 months. Although PSA relapse was observed at 31 months, his serum PSA level was maintained below 2.0 ng/mL with ethinylestradiol for 27 months. However, as shown in Fig. 1, his PSA level rebounded to more than 10 ng/mL, and bone scan revealed expansion of the right pelvic bone metastases 93 months after the introduction of ADT.

### Introduction of docetaxel, enzalutamide, cabazitaxel, and radium-223

We started docetaxel (75 mg/m2) as the second-line therapy, which decreased his PSA level to 1.70 ng/mL after six cycles. However, CT scan showed metastatic progression in the right pelvic bone and new metastases in the sacrum, pubis, and right lung. As the third-line therapy, enzalutamide was administered, but the treatment was halted after 7 months because it failed to decrease the patient’s PSA level. Thus, as the fourth-line therapy, 25 mg/m2 of cabazitaxel (CBZ) was introduced, and it initially decreased his PSA level to 4.86 ng/mL. However, PSA relapse was observed after 4 months and radium-223 (Ra-223) was added to the regimen. As shown in Fig. 1, the combination of 10 cycles of CBZ and 6 courses of Ra-223 could not suppress the elevation of PSA levels or diminish bone metastases as revealed by bone scans.

### Second biopsy and platinum therapy

Second prostate biopsy was performed 132 months after the primary diagnosis in search for possible neuroendocrine differentiation, which was suspected because of the high serum neuron-specific enolase (NSE) level of 30.7 ng/mL. Conversely, no viable tumor cells were detected in the primary lesion. Instead of CBZ, abiraterone was introduced as the fifth-line therapy, and radiation therapy (37.5 Gy/15 Fr) was given to the pelvic bone metastases, leading to a dramatically decreased PSA level from 81.77 to 19.9 ng/mL. However, the patient’s PSA level rose again after 6 months, and new metastases in the L4/5 vertebrae were detected in the bone scan. Based on the increased serum NSE level (30.9 ng/mL), we again suspected neuroendocrine differentiation and decided to introduce the sixth-line therapy comprising of an 80% dose of cisplatin (CDDP) and a 100% dose of etoposide (VP-16) along with radiation therapy to the L5 vertebrae, sacrum, left pubis, and right femur, which resulted in decreased PSA and NSE levels. Although bone scans revealed decreased accumulation in the bone metastases, the patient’s PSA level began to increase 11 months after the initiation of CDDP + VP-16 therapy. Four cycles of CBZ were administered as the seventh-line therapy, and four cycles of carboplatin (CBDCA) and irinotecan (CPT-11) were continuously introduced as the eighth-line therapy. However, the patient’s PSA level further increased, and expansion of lymph node metastases was detected on CT scan.

### Biopsy to lymph node metastases and spinal decompression surgery

**Fig. 2:**
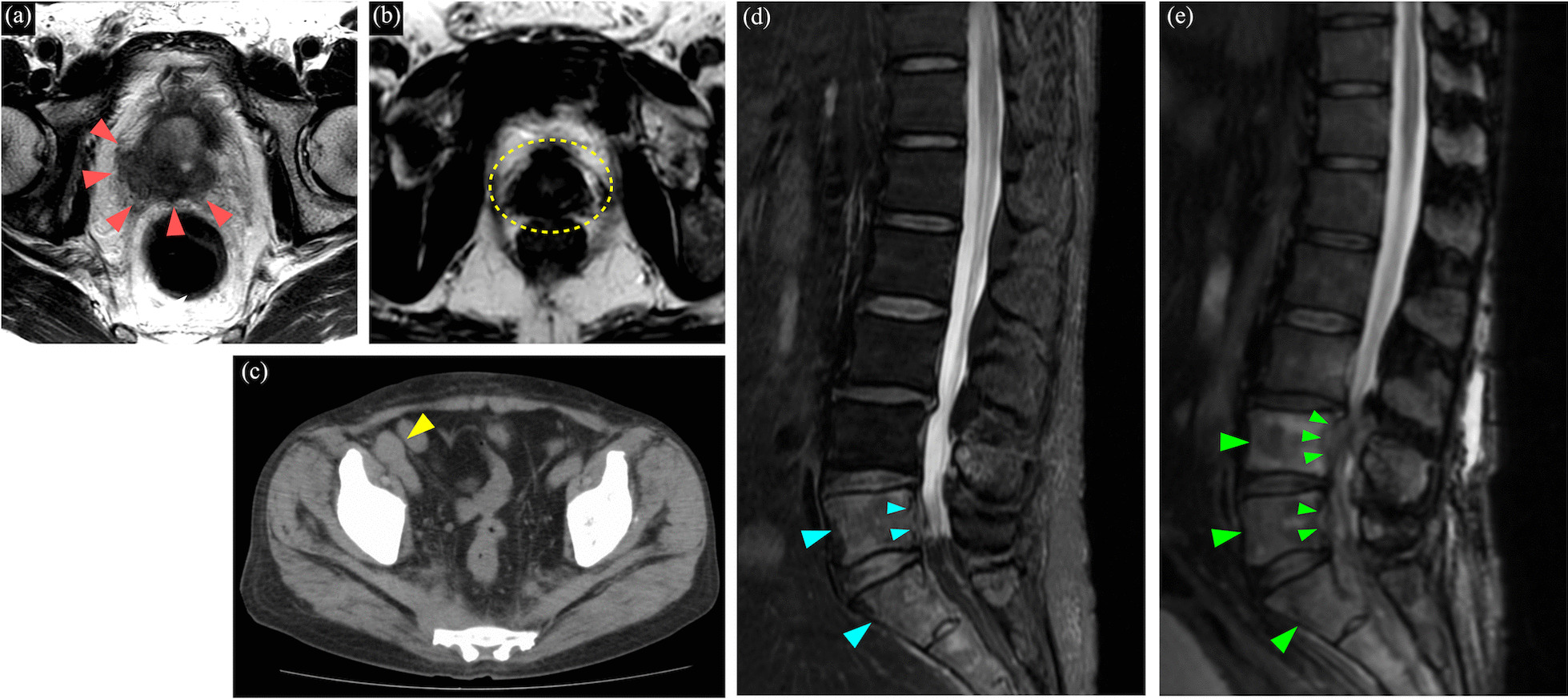
Primary and metastatic lesions during the treatment course. **a** T2WI of MRI showed a prostate tumor invading the right seminal vesicle at the primary diagnosis. **b** MRI showed that the primary lesion had shrunk after androgen deprivation therapy (ADT) and cabazitaxel (CBZ) therapy. The second biopsy revealed total elimination of the variable tumor. **c** T1WI of MRI with Gd enhancement showed the enlarged right inguinal lymph node, which was biopsied for whole-exome analysis and underwent WGD. **d** T2WI of lumbar MRI performed after CBZ showed diffuse metastases in L5/S1 vertebrae. The metastatic tumor in L5 was reaching toward the epidural space. **e** Lumbar MRI performed 2 years later showed the spreading of metastases to L4 vertebrae and spinal cord compression at the level of L4/L5, causing leg paralysis

**Fig. 3:**
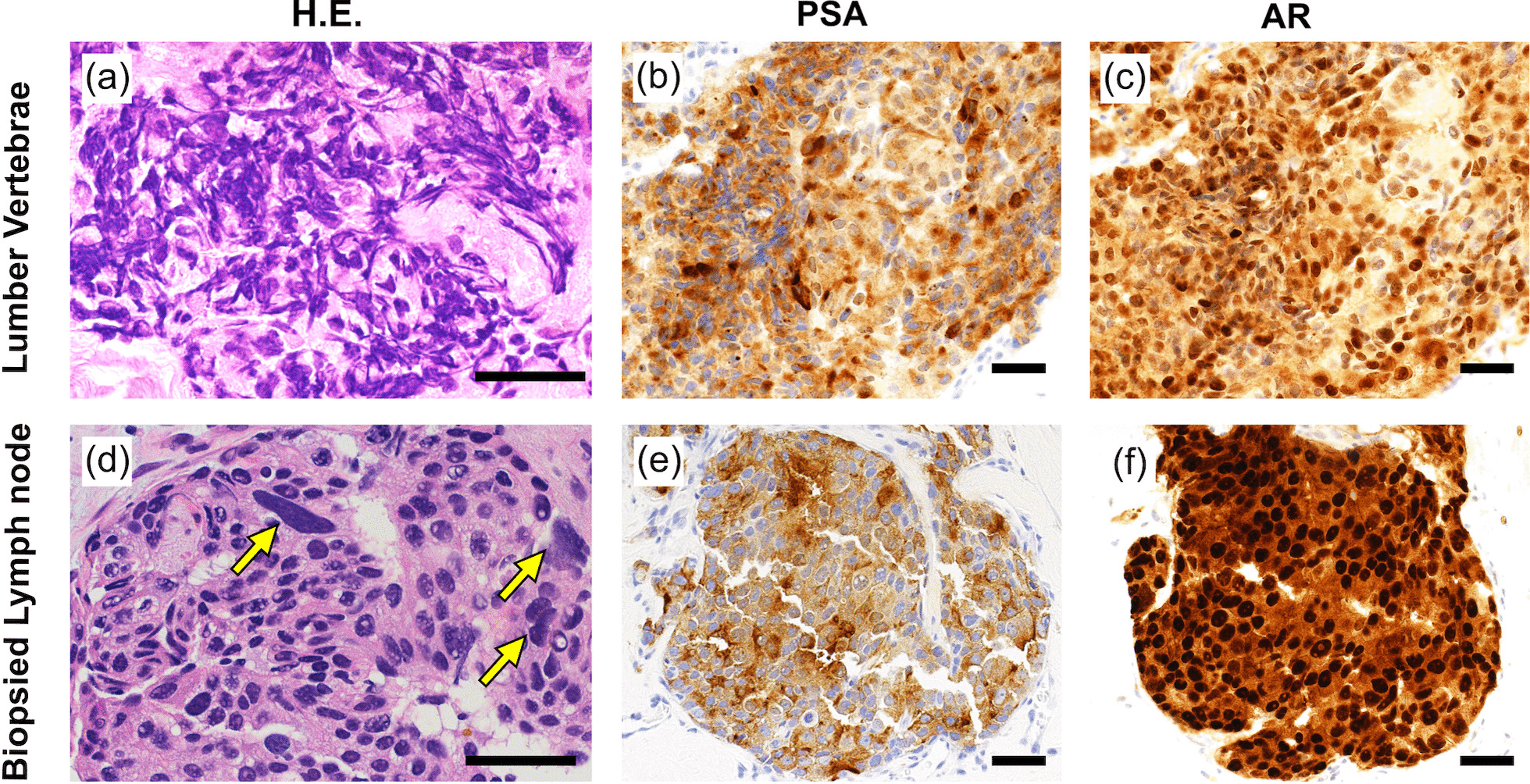
Histology and immunohistochemistry of the specimens collected from metastatic lesions. **a**–**c** Metastatic tumor in the lumbar vertebrae. **d**–**f** Biopsied specimen from the right inguinal lymph node. **b**, **e** Immunochemistry for PSA and **c**, **f** androgen receptor. **d** Tumor cells in the lymph node metastasis, which showed WGD, had polymorphic and larger nuclei. Bars indicate 50 μm

We performed a CT-guided biopsy on the swollen right inguinal lymph node to study the tumor’s pathological characteristics (Fig. 2). The biopsied lymph node contained adenocarcinoma. Immunohistochemical analysis revealed that this tumor was positive for PSA and androgen receptor (AR) and negative for chromogranin and synaptophysin, suggesting that the metastatic lesion unlikely had neuroendocrine differentiation (Fig. 3). Thus, CBZ therapy was restarted as the ninth-line therapy, but after 2 days, the patient manifested sudden muscle weakness of both legs, showing a manual muscle test (MMT) result of 2/2, particularly in the gastrocnemius muscles. Lumbar MRI showed that the metastatic tumor in L4 and L5 vertebrae and sacrum were extending to the epidural space and compressing the spinal cord. Thus, spinal decompression surgery was performed, and the MMT of both legs subsequently recovered to the normal level. The immunohistochemical analysis of the resected bone metastases identified the PSA and AR positive cancer cells, similar to the result of lymph node metastases (Fig. 3). After recovering from the spinal surgery, four cycles of CBZ were administered, and the patient’s PSA level remained consistent at 100 ng/mL.

### Analysis of whole-exome sequencing and microsatellite instability

**Fig. 4:**
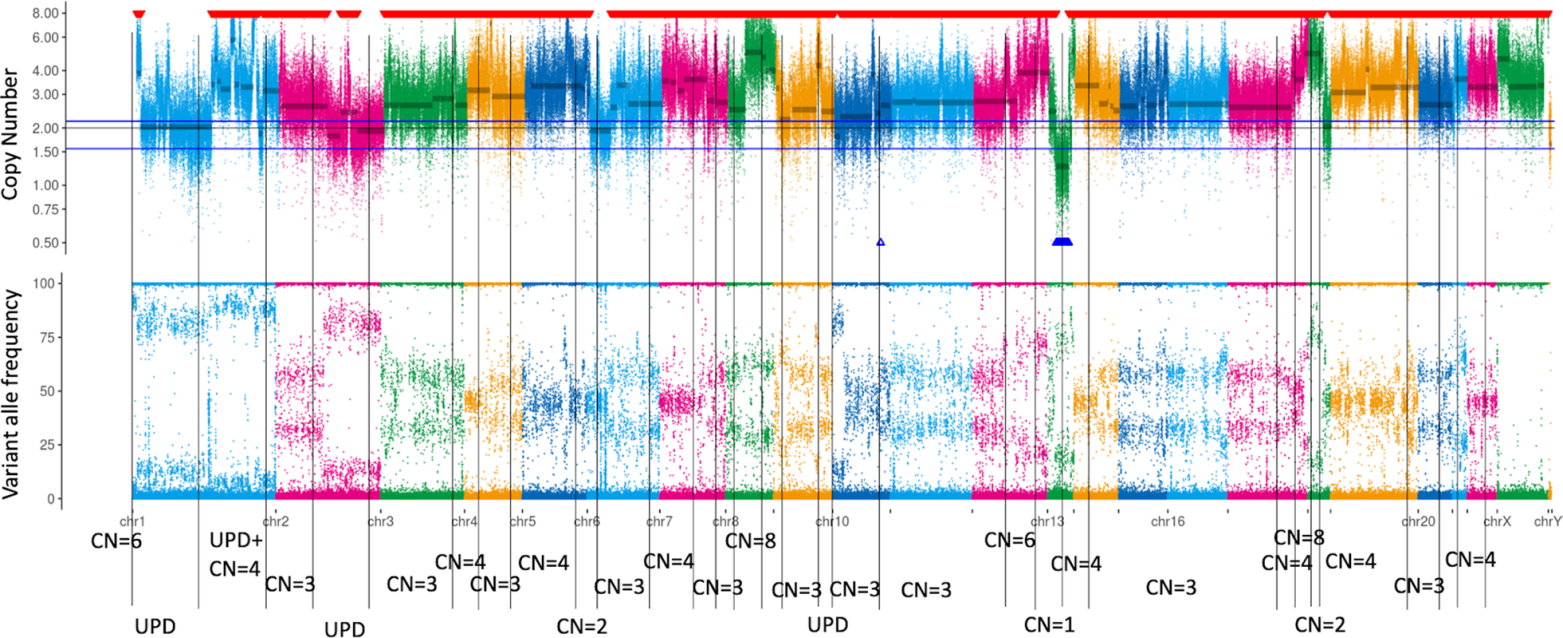
DNA copy number variations and alle variant frequencies of all the chromosomes detected via whole-genome sequencing. Most of the major copy numbers of each chromosome were more than two, which indicated that the tumor had undergone WGD

Seeking other possible therapies, the patient agreed to undergo whole-exome sequencing (WES) using the PleSSision Exome test and samples were collected via lymph node biopsy. WES through the PleSSision Exome test analyzes both tumor and germline DNA. As it is a tumor-normal matched paired test, genomic deoxyribonucleic acid (DNA) was extracted and purified from tumor tissues and from peripheral blood mononuclear cells obtained from the patient. This examination can detect both single-nucleotide variants (SNVs) and copy number variants (CNVs) of 19,296 genes, enabling a wide range of diagnoses. The tumor mutation burden (TMB) is also calculated via WES by the number of SNVs, insertions, and deletions per mega-base in coding regions. The analysis revealed mutations in CDK12 (p.G101Dfs*23), FOXA1 (p.R261C), MACC1 (p.M480L), and HIP1 (p.Q265E). There were no mutations or amplifications of AR, TP53, PTEN, SPOP, MYC, or PI3K pathway and WNT pathway genes. DNA repair genes except for CDK12 had no alterations. Copy number amplifications were observed in 85 driver genes, including CDK12. Detailed information about gene alterations is presented in Additional file 1: Table S1. Regarding the definition of TMB, non-synonymous SNV counts ≥ 150 were defined as likely hypermutated, and non-synonymous SNV counts ≥ 200 were defined as hypermutated. The tumor had 155 somatic non-synonymous SNVs, indicating a relatively high TMB. Interestingly, copy number variation (CNV) sequencing showed twice or more copy numbers in almost all chromosomes, indicating a phenomenon called whole-genome doubling (WGD). Figure 4 illustrates the CNV status on each chromosome. To administer PD-1 inhibitors, we examined the same biopsy specimen for microsatellite instability (MSI), and a negative MSI result was obtained. PARP inhibitor, which is a candidate for CDK12 mutation-positive cancer, had not yet been covered by Japan’s health insurance; thus, he continued CBDCA + CPT-11 therapy as the tenth-line therapy for 3 months, resulting in a gradually increasing PSA level and a generally good performance status.

### Methods

We used OLYMPUS IX83 (microscope) and OLYMPYS UPLFLN10X, UPLFLN20X (objective lens) and OLYMPUS DP80 (CCD camera) for histological analysis of the specimens. The images were captured via OLYMPUS cellSens Dimension 1.14. The resolutions of the original images were all 72 dpi, while the resolutions of processed images shown in the Fig. 3 were increased to 300 dpi via Vectornator 4.2.2 (Linearity GmbH, Berlin).

## Discussion and conclusion

Since the mechanism of the AR axis was revealed, metastatic castration-resistant prostate cancer (CRPC) has been conventionally treated with AR pathway inhibitors such as abiraterone and enzalutamide, or taxane-based chemotherapies. Recently, a new treatment approach using whole-exome sequencing for advanced prostate cancer has been initiated. In this analysis, approximately 20,000 of all the genes of the human genome were surveyed to detect clinically important gene mutations and the status of TMB and MSI [1]. In addition, several detected somatic mutations can be targets of chemotherapies and immune checkpoint inhibitors beyond cancer types. Patients with prostate cancer exhibiting deleterious germline or somatic homologous recombination repair (HRR) gene mutations such as BRACA1/2 and ATM mutations are candidates for olaparib, an inhibitor of PARP1/2 pathways. In the PROfound study, which is an open-label, multicenter randomized trial targeting 256 patients with prostate cancer with such gene mutations, the olaparib cohort exhibited better radiographic progression-free survival and overall survival (OS) than the enzalutamide or abiraterone cohort [4].

In the present case, WES revealed a relatively high TMB, CDK12 mutation, and the presence of WGD. The definition of WGD is varied in previous reports, while Peter Priestley et al. defined WGD as a major allele ploidy greater than 1.5 on 50% of at least 11 autosomes as the number of duplicated autosomes [5]. The method of judging the WGD status from WES or targeted clinical sequencing in a matched tumor and normal blood specimens has not been established. The presence of genome doubling in the tumors was identified using independent and successive copy number alterations (CNAs). All CNAs were estimated from purity-corrected genome-wide integer copy number obtained by WES, which counts maternal and parental alleles according to the sequencing coverage and genotypes of germline single-nucleotide polymorphisms. WGD is relatively common in cancer genomes. In Craig M. B. et al.’s report, 28.2% of 9692 patients with various advanced cancers had WGD [2]. Although nearly 20% of patients with prostate cancer showed WGD [2], no such case has ever been reported in the Japanese population. Furthermore, while WGD is known to provide cancer cells with an advantage in survival, its mechanism remains unclear. According to one theory, WGD is selected during cancer cell evolution because it buffers the effects of post-WGD deleterious mutations by providing more mutation-free genes [6]. A previous study analyzed the genome profiles of 783 patients with prostate cancer; while only 4% of the primary prostate cancers of 513 patients presented WGD, 46% of the metastatic tumors of 270 patients with CRPC did, most of which were heavily treated with AR pathway inhibitors or taxane-based chemotherapy [2]. This report is similar to our case, in which WGD was observed in a patient with CRPC who was intensely treated with a series of AR antagonists and chemotherapies for more than 13 years.

The histological characteristics of the metastatic tumor in our case were unique. Many of the tumor cells contained relatively large, polymorphic nucleus, as shown in Fig. 3f. Elizabeth G et al. studied 235 adult sarcomas and revealed that increased nuclear size and polymorphism strongly correlated with the presence of nuclear WGD [7]. In our case, the enlarged nucleus of the metastatic tumor may reflect the high complexity of the chromosomes.

CDK12 is a cyclin-dependent kinase that contributes to DNA repair by regulating homologous recombination DNA repair genes such as ATR, BRACA1, FANCI, and FANCD2. CDK12 mutation occurs in 4%-11% of prostate cancer cases [8], and several retrospective studies have demonstrated that CDK12 mutation in prostate cancer is associated with aggressive clinical features, including higher prevalence of high-risk tumors, shorter time to castration resistance, shorter time to PSA progression, and shorter overall survival [8–10]. As it is reported that CDK12 deficiency serves as a biomarker of PARP inhibitor sensitivity in patients with ovarian cancer [11], patients with prostate cancer having CDK12 alterations are expected to exhibit good responses to PARP inhibitors. On the contrary, several studies have revealed that these patients have a shorter time to PSA progression than those with other mutations [8], or a poorer PSA response and progression-free survival compared to those treated with PD-1 inhibitors [9]. Although merely 33.3% of patients displayed PSA responses in one of these studies, immune checkpoint therapy is considered another promising candidate for the treatment of patients with CDK12-mutated prostate cancer. The finding that CDK12-deficient prostate cancers express extremely high levels of fusion-induced neoantigens implies the possibility of immunotherapy sensitivity [12]. In our case, PD-1 inhibitors such as pembrolizumab could be candidates for the treatment after the ninth-line CBZ therapy.

Another interesting aspect of our case is that the patient had survived for 8 years with maintained performance status after CRPC diagnosis, probably because of the successful local therapy. While Ra-223 contributed to better OS in patients with mCRPC as shown in ALSYMPCA study [13], the relationship between conventional external beam radiotherapy (EBRT) for metastases and better OS is unknown. In our case, each EBRT for pelvic bone metastases resulted in an acute decrease of serum PSA level and improvement in bone scintigraphy (Fig. 1). A good control of metastatic lesions might have suppressed cancer progression and achieved a remarkably long survival.

In conclusion, this case report presents the first Japanese case of metastatic CRPC that underwent WGD after a series of taxane- and platinum-based chemotherapy. Although the clinical significance of WGD itself in prostate cancer remains uncertain, further development in genome sequencing may reveal its importance and clinical application.

## Supplementary Information

**Additional file 1: Table S1**. Detailed information about gene alterations in COSMIC Cancer Gene Census Tier1 or Tier2 (728 genes).

## Data Availability

The datasets generated and analysed during the current study are available in the Genome Sequence Archive for Human repository (https://ngdc.cncb.ac.cn/gsa-human/browse/HRA001553).

## Abbreviations

WES: Whole-exome sequencing
PSA: Prostate-specific antigen
NSE: Neuron-specific enolase
ADT: Androgen deprivation therapy
AR: Androgen receptor
MMT: Manual muscle test
CRPC: Castration-resistant prostate cancer
WGD: Whole-genome doubling
TMB: Tumor mutational burden
MSI: Microsatellite instability
SNV: Single-nucleotide variant
CNV: Copy number variant
